# Results for Adults with Asymmetric Hearing Loss Who Receive a Cochlear Implant

**DOI:** 10.3390/audiolres16040102

**Published:** 2026-07-23

**Authors:** Alexandra Rousset, Sylvia Tari, Rod Hollow, Raoul Wills, Richard C. Dowell

**Affiliations:** 1 Cochlear Implant Clinic, The Royal Victorian Eye and Ear Hospital, 32 Gisborne St, East Melbourne, Melbourne, VIC 3002, Australia; 2 HEARnet Clinical Studies, HEARnet Ltd, 550 Swanston St, Carlton, Melbourne, VIC 3053, Australia; 3 Department of Audiology and Speech Pathology, University of Melbourne, Grattan St, Parkville, Melbourne, VIC 3010, Australia

**Keywords:** cochlear implant, unilateral hearing loss, SRT, counselling, localisation, tinnitus, quality of life

## Abstract

**Objectives**: To collate subjective and objective outcome measures from cochlear implant recipients with asymmetric hearing loss. **Methods**: Forty-one CI recipients with asymmetrical hearing loss completed spatially separated speech in noise testing, speech in quiet testing, localisation testing, and tinnitus and quality of life questionnaires (SSQ and IOI-HA) pre-operatively and at 3, 12 and 24 months post-operatively. The results were used to generate a tool to help clinicians guide potential recipients through the counselling process. **Results**: Significant improvements in spatially separated SRT, localisation, tinnitus and the SSQ were observed for the group, with significant individual variability. CVC phoneme scores were not significantly different from those observed for CI recipients with bilateral hearing loss. The rates of non-use were consistent with those in the literature and were higher than for recipients with bilateral hearing loss. There was a significant correlation between PTA in the contralateral ear and localisation, and those recipients with a shorter duration of hearing loss were more likely to be satisfied with their CI. Discussion: Cochlear implantation for patients with asymmetrical hearing loss has the potential to offer improvements in head-shadow effects, localisation and tinnitus, with subjective measures also showing significant improvements post-operatively. There exists a large amount of individual variability across the group, however, which makes pre-operative counselling problematic for this patient cohort. **Conclusions**: The use of the study-generated counselling tool by clinicians may help patients make a more educated decision to proceed with implantation based upon realistic expectations of possible post-operative outcomes.

## 1. Introduction

The presence of single-sided, or asymmetric, hearing loss can pose significant auditory and psychosocial issues for an individual. Asymmetric hearing loss can detrimentally impact sound localisation and the binaural squelch effect, introduce a head-shadow effect, increase listening effort and may also involve the addition of tinnitus [1,2]. Historically, single-sided, or asymmetric, hearing losses have been treated with a CROS (Contralateral Routing of Signal) hearing aid or a bone-anchored hearing aid (BAHA). Whilst these devices are successful in overcoming the head-shadow effect and may reduce listening effort, the literature has shown limited benefit with regards to sound localisation and tinnitus suppression, as binaural hearing is still deficient due to the auditory stimulation being routed to the better hearing ear [1,3,4].

Many studies have shown that cochlear implants (CIs) have the ability to restore auditory function in a deafened ear, thereby improving speech recognition in noise, sound localisation, and tinnitus [1,5,6,7]. Improved speech perception in various noise configurations has been documented by many studies, reinforcing the importance of binaural hearing in challenging listening environments and therefore supporting the provision of CIs for patients with single-sided hearing loss [2,8,9,10].

Sound localisation is dependent on the discrimination of interaural level and timing differences. Arndt et al. compared outcomes of CI, BAHA and CROS for localisation using a seven-speaker set up and found a significant improvement in localisation with the CI, when compared to the unaided and the BAHA and CROS conditions. Significant improvements in post-operative localisation have been demonstrated by many studies [2,3,11].

Van de Heyning and colleagues have suggested that one possible cause for tinnitus could be the generation of a percept due to the absence of any auditory stimulation, which is a potential risk in single-sided hearing loss. It was therefore hypothesised that stimulation of the deaf ear and subsequently the auditory cortex, via provision of a CI, may have the potential to ease such tinnitus, or at least the awareness of the tinnitus [2,12]. Various studies have demonstrated easing of tinnitus after cochlear implantation for single-sided hearing loss, with rates of improvement at 80 and 95% [1,12].

The duration of hearing loss in the ear to be implanted is a well-accepted predictor of CI outcomes for postlingually deafened adult recipients [13]. Many studies have suggested that the duration of unilateral hearing loss prior to implantation also has the potential to influence outcomes, and it is generally acknowledged that a shorter duration of deafness may lead to better results [14,15,16]. Tavora and Boisvert investigated cochlear implant outcomes for five patients with long-term unilateral deafness, ranging from 27–40-year durations [17]. The results for speech perception and subjective reports collected at 12 months post-implantation indicated scores in a similar range as patients with bilateral hearing loss, which may reflect the ongoing integrity of the bilateral auditory pathways via monaural stimulation in the case of unilateral hearing loss.

Perhaps the most sensitive measure of real-life patient benefit for CI recipients with asymmetric hearing loss would relate to the hours of implant use. Generally, the rate of non-use among CI recipients is low, with two large studies reporting non-use rates of 1–10% and 2.8%, respectively [18,19]. Rates of non-use are often assumed to be higher for recipients with unilateral deafness, but this does not always seem to be the case. A large study by Deep et al. followed 53 CI patients with single-sided deafness. They found that after three years, the non-use rate was 3.7%, which was significantly better than the non-user rate reported in patients with BAHA of around 14–19% [20,21]. Full-time device use after one year has been reported in numerous studies [1,12,22], with data logging showing a similar time on air to bilaterally deaf CI users [23]. Longer-term patient follow-up may be a more accurate reflection of real-life benefit. A long-term study by Tavora-Vieira investigated device use over 10 years for 114 patients and found a non-use rate of 4.4%, which is similar to those rates reported by Vlastarakos [2,24].

Non-use, or limited use, of the implant for this patient group is more likely a reflection of the perceived changes to the recipient’s quality of life post-surgery, rather than objective results obtained on speech perception testing. The primary aim of medical intervention is to improve a patient’s quality of life, and in terms of cochlear implantation, this means not only improving a person’s ability to hear but also positively impacting their self-esteem, social interactions and daily functioning [25]. Whilst many studies have shown an improvement in results on the Speech, Spatial and Qualities of Hearing Scale Questionnaire (SSQ) post-implantation [8,11], it has also been shown that stress levels, depression and anxiety have the potential to be improved [26].

Whilst there are a multitude of studies demonstrating benefits gained by offering CIs to patients with asymmetric hearing loss, common themes remain: the variability in outcomes and acceptance of the device, and higher rates of non-use than those documented for patients with bilateral hearing loss. This raises the difficult task for clinicians of ensuring that patients are adequately counselled about expectations prior to surgery. The aims of the current study were to collate subjective and objective outcome measures from a large group of patients with asymmetric hearing loss who proceeded with cochlear implantation at the Melbourne Cochlear Implant Clinic. The distribution of the results was analysed with the purpose of offering prospective recipients an indication of the chance of improvement post-surgery, enabling them to make fully informed decisions about proceeding with implantation based on realistic expectations of outcomes.

## 2. Participants

Ethics approval for this project was granted by the Royal Victorian Eye and Ear Hospital Human Research Ethics Committee (project 15/1229H). Forty-one adults with asymmetric hearing loss from the Melbourne Cochlear Implant Clinic at The Royal Victorian Eye and Ear Hospital were recruited for this study. All participants signed a study consent form approved by the HREC committee. For the purpose of this study, asymmetric hearing loss was defined as a four-frequency pure tone average (PTA) greater than 60 dB in the ear to be implanted and an average PTA less than 50 dB in the contralateral ear. Twenty-one participants had true SSD, categorised as PTA in the contralateral ear of less than 20 dB. The duration of hearing loss in the ear to be implanted ranged from 3 months to 21 years, with the exception of one patient with a congenital asymmetric hearing loss, with duration noted as 41*.

The aetiologies of hearing loss included the following: Genetic (*n* = 2), Otosclerosis (*n* = 2), Meningitis (*n* = 2), Trauma (*n* = 2), Labyrinthitis (*n* = 2), Prenatal–structural (*n* = 1), Meniere’s Disease (*n* = 5) and Unknown (*n* = 25). All patients were assessed as audiologically, medically and radiologically suitable for cochlear implantation, as per the standard clinical guidelines [27]. No participant had retrocochlear pathology. Participants underwent extensive counselling to ensure that they had realistic expectations of possible cochlear implantation outcomes.

All participants received either a Nucleus CI512 or CI522 Cochlear implant and were fitted with either a Nucleus 6 or Nucleus 7 sound processor. All participants received standard clinical programming of their sound processor from an experienced audiologist. Refer to Table 1 for recipient demographics.

## 3. Methods

Speech understanding in noise was completed with each recipient pre-operatively and at 3, 12 and 24 months post-operatively using the Australian sentence test in noise [28]. The test is an adaptive speech in noise test used to determine the Speech Reception Threshold (SRT), defined as the signal-to-noise ratio (SNR) for 50% sentence understanding. A spatially separated signal was used for assessment with both the S90 N270 (speech at 90 degrees, noise at 270 degrees) and S270 N90 (speech at 270 degrees, noise at 90 degrees) speaker configurations. Two lists of sentences were used in each condition. A verified hearing aid was permitted to be used in the contralateral ear for those participants who normally wore one. The implanted ear was tested ‘unaided’ in the pre-operative condition and using the CI for all post-operative test points.

Post-operatively, words in quiet were tested at 3, 12 and 24 months using two lists of 50 CVC (Consonant–Vowel–Consonant) words, presented in quiet, audition alone at a level of 65 dB. An Australian male talker was used, and the list of phonemes was balanced. ted via direct audio input to a CP600 sound processor, with the processor itself placed into a calibrated soundproof Otocube with a reference speaker, with a 100 cm coil cable used for attachment to the participant’s implant.

Sound localisation was measured with bursts of pink noise presented from a 13-speaker array over 180 degrees. Two test runs were completed, with each run comprising five presentations from each speaker in random order. Participants were seated facing the middle speaker and selected which speaker they believed the sound originated from. A root mean square (RMS) angular error was calculated for each run.

The Speech, Spatial and Quality of Hearing Scale (SSQ) was administered pre-operatively and at each post-operative assessment point [29]. This questionnaire is designed to assess subjective functional hearing impairment in a variety of everyday complex listening situations, to measure any benefits obtained post-implantation.

The International Outcome Inventory for Hearing Aids (IOI-HA) questionnaire was used to evaluate the participant’s overall satisfaction with their CI at each post-operative point [30]. This is a seven-item questionnaire designed to be applicable in evaluating the general effectiveness of hearing aid treatments.

Pre- and post-operative tinnitus was assessed via the Iowa Tinnitus Handicap Questionnaire [31]. This questionnaire is designed to examine the physical, emotional and social consequences of tinnitus; the hearing ability of the patient; and the patient’s view of their tinnitus.

Cochlear implant use was documented via datalogging collected by the participant’s clinician at their routine mapping sessions that preceded each assessment point.

## 4. Results

### 4.1. Objective Results

#### 4.1.1. Speech Perception

The majority of recipients gained post-operative improvement on the spatially separated SRT. A two-way General Linear Model ANOVA with SRT score as the response, time as a fixed factor and subject as a random factor demonstrated no significant improvement between the 3-, 12- and 24-month post-operative time points (F = 2.04, *p* = 0.138); therefore, the 12-month point was chosen for analysis.

**Figure 1:**
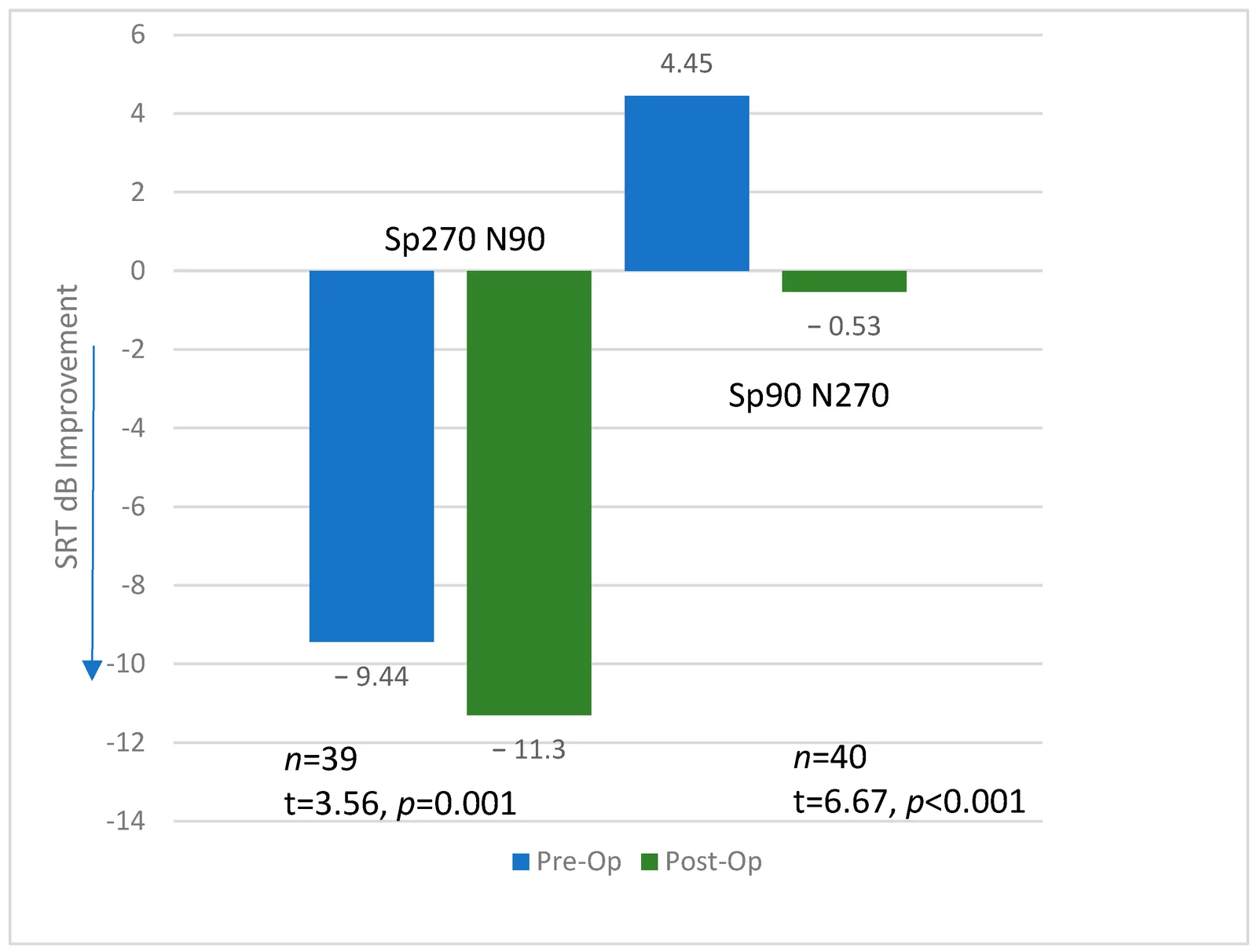
Group SRT improvements from pre-operative to 12 months post-operative.

Figure 1 shows the improvement in the SRT for the entire group from the pre-operative to the 12-month post-operative point. The results have been adjusted for multiple comparisons using the Bonferroni correction, changing the alpha level to 0.025. When speech was presented to the good ear and noise to the poor ear, the mean SRT improved from −9.44 dB to −11.3 dB (t = 3.56, *p* < 0.001) for 39 recipients. This corresponds to a moderate effect size of 0.57 (Cohen’s d) with a confidence interval of 0.803 to 2.92. When speech was presented to the poor ear and noise to the good ear, the mean SRT improved from 4.45 dB to −0.53 dB (t = 6.67, *p* < 0.001) for 40 recipients, corresponding to a strong effect size of 1.05, with a confidence interval of 3.49–18.49. These results remained significant with the outlier removed (t = 3.35, *p* = 0.002, and t = 7.12, *p* < 0.001).

**Figure 2:**
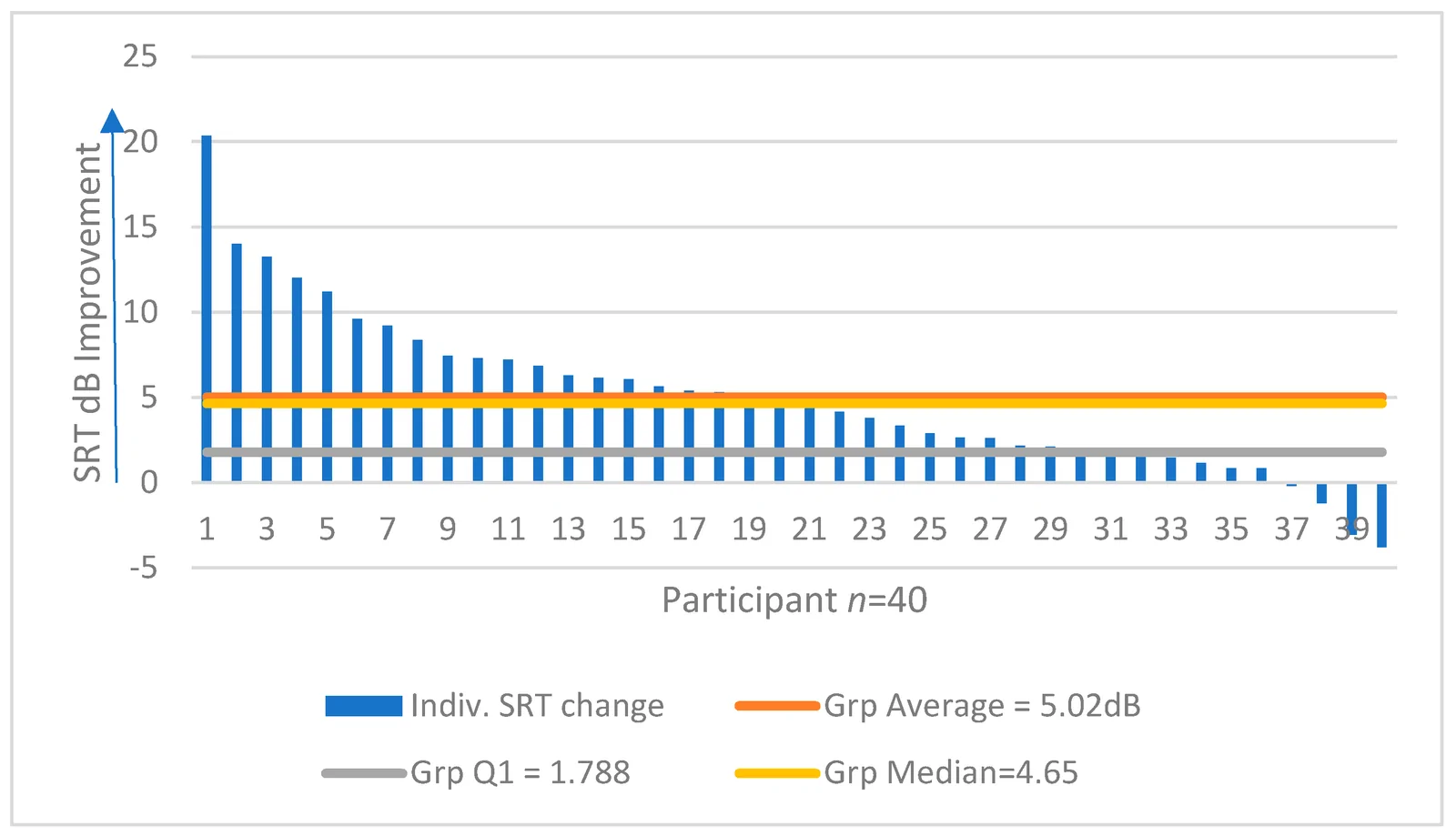
Individual SRT differences from pre-operative to 12 months post-operative (dB).

While a significant improvement was shown for the group, individual variability in SRT was observed. Figure 2 demonstrates the change in the head-shadow effect when speech was presented to the poor ear and noise to the good ear from the pre-operative to 12-month post-operative point for the 40 recipients. There were four recipients who showed a negative result, indicating that their SRT result was poorer at the 12-month post-operative point than it was pre-operatively. Of these four recipients, one did not obtain a significant open set CVC phoneme score post-operatively, but the other three scored 30%, 70.5% and 83% for the phoneme scores at the 12-month point.

The mean post-operative phoneme score for the group was 55.92% at 12 months, which, while not significantly different from the mean score of 65.11% obtained from 385 adult recipients from the Melbourne Cochlear Implant Clinic (t = −1.94, *p* = 0.059, CI −19.58 to −0.21) [27] with an effect size of 0.345, was approaching significance and may indicate a tendency towards a non-significant trend for lower scores. Significant individual variability in post-operative phoneme scores was observed, with four recipients scoring between 0 and 10%.

A two-way ANOVA with phoneme scores as the response, time as a fixed factor and subject as the random factor showed no significant improvement in phoneme scores between the 3-, 12- and 24-month post-operative points (F = 0.86, *p* = 0.429).

A significant moderate correlation was observed between the 12 months SRT score with speech presented to the CI ear and the 12 months CVC phoneme score (r = −0.608, *p* < 0.001), with those patients who performed better on the SRT test also obtaining a higher phoneme score.

#### 4.1.2. Localisation and Tinnitus

**Figure 3:**
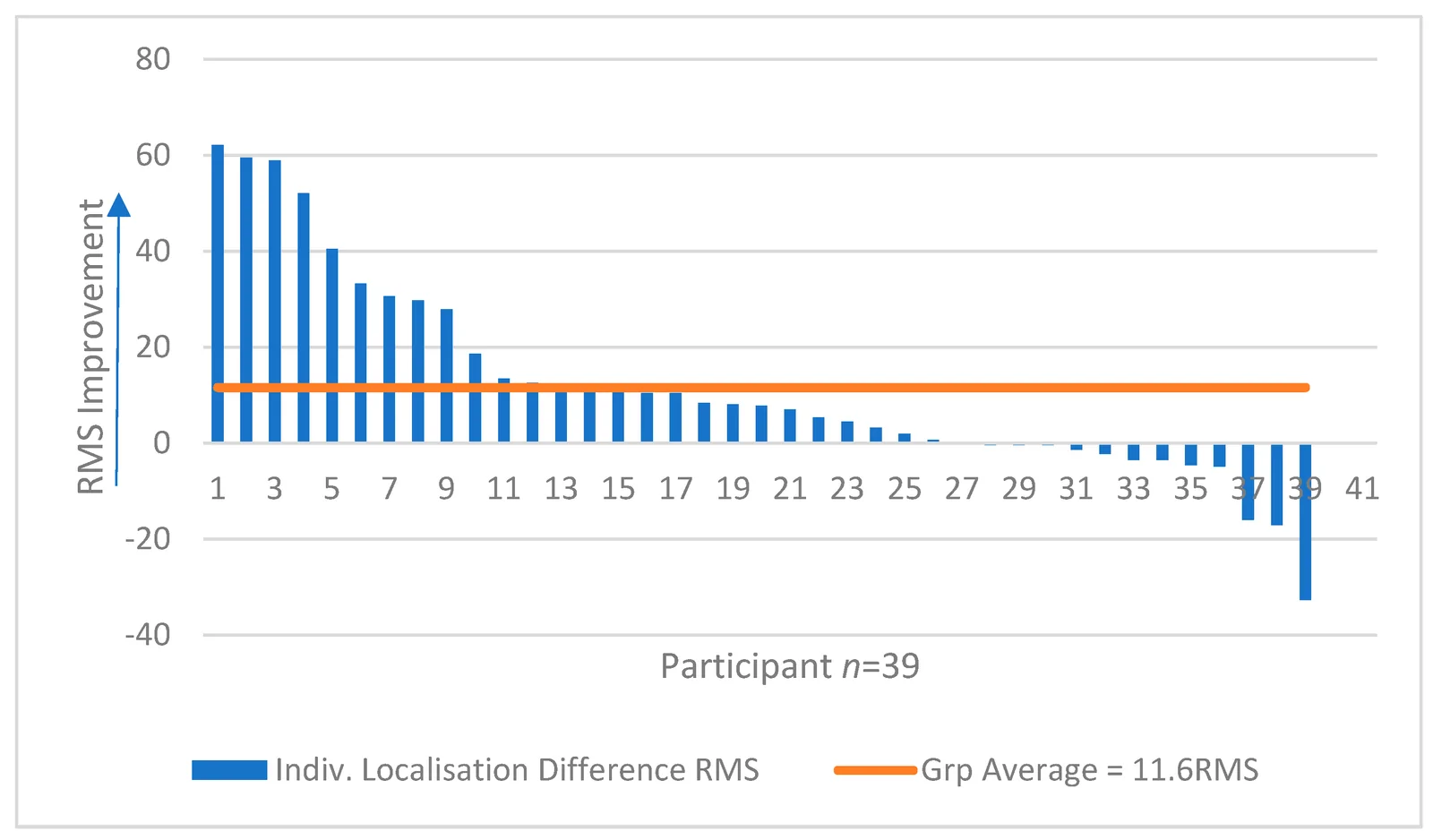
Individual localisation differences in RMS from pre-operative to 12 months post-operative.

A two-way ANOVA showed that there was no significant improvement in the post-operative sound localisation results between 3, 12 and 24 months (F=2.01, *p* = 0.143); therefore, the 12-month point was chosen for analysis. A significant improvement was demonstrated for the group from pre-operative (mean = 42.84 RMS error) to 12 months post-operative (mean = 31.20 RMS, t = 3.44, *p* = 0.001), with a confidence interval of 4.79–18.49 and an effect size of 0.55, but significant individual variability was observed. This result remained significant with the outlier removed (t = 3.45, *p* = 0.001). Refer to Figure 3.

There was a significant, albeit weak, correlation between localisation and speech perception, with those recipients who performed better on the localisation task also obtaining a better score on SRT (r = 0.324, *p* = 0.041) and CVC phonemes (r = −0.379, *p* = 0.021). There was no significant correlation between localisation and tinnitus (*p* = 0.064).

**Figure 4:**
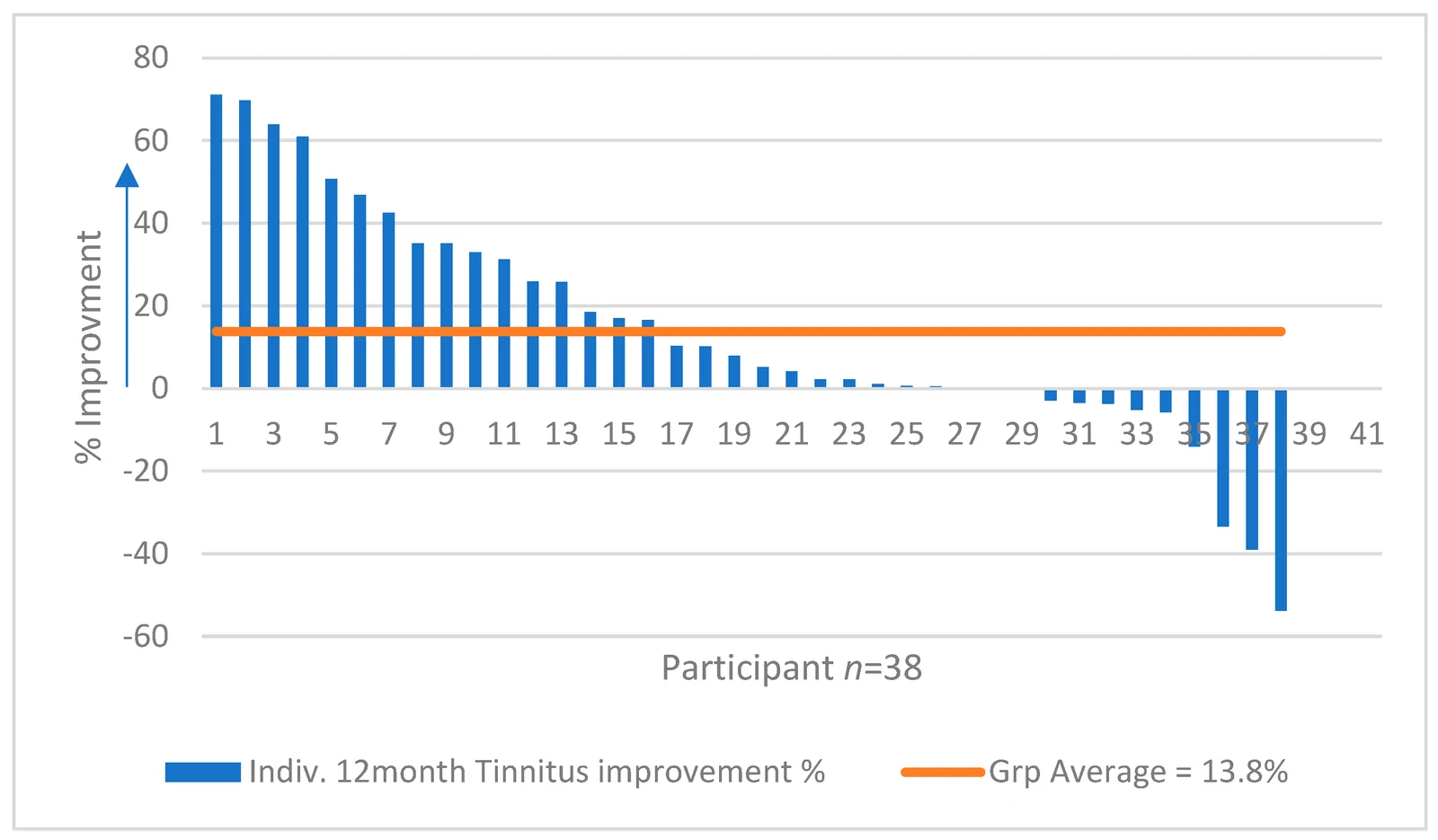
Individual tinnitus difference from pre-operative to 12 months post-operative (%).

A two-way ANOVA showed no significant difference in tinnitus between the 3-, 12- and 24-month post-operative points (F = 0.12, *p* = 0.883). A significant improvement was observed from the pre-operative (mean 34.85%) to the 12-month post-operative point (mean 20.99%, t = 3.01, *p* = 0.005), with a confidence interval of 4.52–23.2 and an effect size of 0.48, again with significant individual variability observed. This result remained significant with the outlier removed (t = 3.02, *p* = 0.005). Refer to Figure 4.

There was no correlation between tinnitus and SRT (*p* = 0.881) or CVC phonemes (*p* = 0.296).

### 4.2. Subjective Results

#### 4.2.1. SSQ: The Speech, Spatial and Quality of Hearing Scale Questionnaire

A two-way ANOVA showed no significant difference in the total SSQ score obtained at 3, 12 and 24 months post-operative (F = 1.10, *p* = 0.341) for the 33 recipients; therefore, the 12 months point was chosen for analysis.

A paired T-test showed significant improvement from pre-operative to 12 months post-operative for the total SSQ score and for each of the three individual domains, with a corrected alpha level of 0.0125. These results remained significant with the outlier removed (*p* < 0.001). Estimation of the error using the standard deviation of the differences (3.9) showed that 14 out of 33 recipients (42%) demonstrated significant improvement in their SSQ scores from pre-operative to 12 months post-operative, 52% showed no significant change, and 6% showed a significant deterioration in scores. Refer to Table 2.

A significant correlation was observed between the 12-month SSQ domain 2 (spatial domain) and the 12-month localisation result (r = −0.535, *p* = 0.001, CI −0.734 to −0.250). A significant correlation was also observed between the 12-month SSQ total score and 12 months localisation (r = −0.502, *p* = 0.002, CI −0.711 to −0.213), with a corrected alpha of 0.0125 for the four comparisons.

There was no correlation between the 12 months SSQ total score and the 12 months CVC phoneme score (*p* = 0.129) or the 12 months SRT score with speech presented to the poor ear (*p* = 0.06). There was no correlation observed between the pre-operative SSQ score and the duration of hearing loss (*p* = 0.872) or between the improvement in the SSQ and the duration of hearing loss (*p* = 0.395).

A significant correlation was observed between the 12 months SSQ total score and the 12 months tinnitus score (r = −0.435, *p* = 0.007), with those patients reporting more severe tinnitus scoring lower on the SSQ. There was also a significant correlation between the SSQ and the IOI-HA (r = 0.479, *p* = 0.006), with those recipients reporting greater benefits on the SSQ also reporting greater overall satisfaction on the IOI-HA.

#### 4.2.2. IOI-HA

A two-way ANOVA showed no significant difference in the IOI-HA total score obtained at 3, 12 and 24 months post-op (F = 2.29, *p* = 0.139); therefore, the 12 months point was chosen for analysis. The adjusted alpha level, correcting for multiple comparisons, was 0.0085.

A significant correlation was observed between the IOI-HA total score and the SSQ total score (r = 0.563, *p* = 0.001), the SRT score with speech presented to the implanted ear (r = −0.482, *p* = 0.005) and sound processor use (r = 0.515, *p* = 0.003). No significant correlations were observed between the IOI-HA total score and the CVC phoneme score (*p* = 0.105), localisation (*p* = 0.699) or tinnitus (*p* = 0.325).

The IOI-HA results were split into two factors: Factor 1 included questions 1, 2, 4 and 7 representing introspection on the hearing device, and Factor 2 included questions 3, 5 and 6, reflecting the influence of the hearing device on the recipient’s interactions with the outside world [30].

Factor 1 (CI introspection) was significantly correlated with the 12 months CVC phoneme score (r = 0.365, *p* = 0.04), SRT (r = −0.226, *p* = 0.001), the SSQ (r = 0.511, *p* = 0.003) and device usage (r = 0.468, *p* = 0.007). There was no significant correlation with localisation (*p* = 0.909) or tinnitus (*p* = 0.233).

Factor 2 (CI interactions) was significantly correlated with the SSQ (r = 0.514, *p* = 0.003) and device usage (r = 0.468, *p* = 0.007). Factor 2 was not significantly correlated with the CVC phoneme score (*p* = 0.698), SRT (*p* = 0.213), localisation (*p* = 0.414), or tinnitus (*p* = 0.748).

#### 4.2.3. Sound Processor Use

A two-way ANOVA showed a significant decrease in sound processor use over time (F = 3.48, *p* = 0.036). Twenty-five out of forty-one recipients (or 60.9%) wore their sound processor for eight hours a day or more at the 3-month point, as measured from Custom Sound programming software version 5.0 at their corresponding mapping appointment. Twenty-three (56%) wore it eight hours or more at the 12-month point. At the 24-month point, 18 of 36 recipients (50%) wore their processors for eight hours or more. Twenty-four-month data were not obtained for five recipients due to the COVID pandemic and recipients being reluctant to come to appointments. The IOI-HA was returned by two of these recipients, who both had selected that they wore their processor ‘more than eight hours per day’. These data points were not included in the 24-month analysis due to their subjective nature.

All recipients wore their processor for more than one hour per day at the 3-month assessment point. At the 12-month point, three recipients wore their processors for less than one hour per day (7%), and these recipients were classified as non-users. The average daily use for all 41 recipients at the 12-month point was 8.29 h. The average daily use for the 3 non-users was 0.43 h, and it was 8.7 h for the remaining 38 recipients.

At the 24-month point, five recipients wore their processor for less than one hour per day (13%). Two of these recipients scored 0% on CVC phonemes, but the other three recipients scored 38%, 70% and 32% on phonemes. The group average for all 36 recipients with data logging available at the 12-month point was 7.99 h. The five non-users had an average daily use of 0.28 h, while the remaining 31 recipients had an average daily use of 8.9 h.

The study participant with congenital onset of asymmetric hearing loss wore their speech processor for 10 h per day at the 3-month point and 9.7 h at the 12-month point, with use dropping to 1.5 h at the 24-month point.

Refer to Table 3 for sound processor use over time.

Sound processor usage was significantly correlated with improvement in SRT from pre-operative to 12 m post-operative, with those recipients who gained the most improvement wearing their processor longer each day (r = 0.463, *p* = 0.005), with an adjusted alpha level of 0.01. A significant correlation was observed for the IOI-HA, with those recipients who wore their processor longer reporting better overall satisfaction with the device (r = 0.462, *p* = 0.008, CI 0.135 to 0.698).

No significant correlations were found between processor usage and the absolute 12-month SRT score (r = 0.341, *p* = 0.031), CVC phoneme score (*p* = 0.107), the SSQ (*p* = 0.440), localisation (*p* = 0.623) or tinnitus (*p* = 0.993).

#### 4.2.4. Effect of the Duration of Hearing Loss

Regression analysis was performed with each outcome measure against the duration of hearing loss in the implanted ear, with duration as a continuous predictor and subject as a categorical predictor.

The duration of loss was not significantly correlated with 12 months post-operative SRT with speech presented to the implanted ear (*p* = 0.15), with 12 months tinnitus (*p* = 0.936) or with the 12 months localisation (*p* = 0.643). There was also no significant correlation with the 12 months IOI-HA (*p* = 0.109), 12 m SSQ (*p* = 0.509) or the 12 months data usage (*p* = 0.987).

A significant correlation was observed with the 12 months CVC phoneme score, with those subjects with a longer duration of hearing loss obtaining a poorer score (r = −0.459, *p* = 0.004, CI −0.682 to −0.159). This correlation remained significant with the outlier removed (r = −0.388, *p* = 0.019).

The group of subjects were divided into those with a duration of hearing loss of 5 years or less and those with a duration of hearing loss greater than 5 years, and two-sample *t*-tests were performed for each outcome measure, with a corrected alpha level of 0.007.

There was no significant difference between the two groups for SRT (*p* = 0.144), CVC phonemes (t = −2.25, *p* = 0.04), localisation (*p* = 0.877), tinnitus (*p* = 0.907), SSQ (*p* = 0.512), or sound processor usage (0 = 0.240). There was a corrected, non-significant trend towards higher IOI-HA scores for those recipients who had a duration of hearing loss less than 5 years.

#### 4.2.5. Effect of PTA in Contralateral Ear

Regression analysis was performed with each outcome measure against the PTA in the contralateral ear, with PTA as a continuous predictor and subject as a categorical predictor, with a corrected alpha level of 0.0056.

No significant correlation was observed with SRT (*p* = 0.024), CVC phoneme score (*p* = 0.05), tinnitus (*p* = 0.192), the SSQ (*p* = 0.044) the IOI-HA (*p* = 0.569) or data usage (*p* = 0.972).

A significant correlation was observed with localisation (*p* = 0.002, CI 0.196 to 0.687), with a moderate effect size of 0.478, where those subjects with a better PTA in the contralateral ear scored better on the localisation tasks.

#### 4.2.6. Possibility of Improvement Post-Surgery

The current Melbourne Cochlear Implant Guidelines for patients with bilateral hearing loss are set such that prospective patients can expect a 75% chance of improvement in speech perception post-implantation [27]. If these same guidelines are applied when analysing the distribution of speech in noise improvement post-implantation for the group of participants in the current study, the first quartile score is 1.788 dB, that is, 75% of the group improved their SRT by more than 1.788 dB. If overcoming the head-shadow effect is the patient’s primary goal for implantation, and they scored less than a 1.788 dB improvement in spatially separated SRT with their BAHA or CROS trial, then there is a 75% chance that a cochlear implant would provide greater head-shadow relief than the BAHA or CROS, based on the first quartile of the SRT improvement distribution in our cohort. This figure of 1.788 was rounded up to 2 dB for ease of use.

For this particular patient cohort, speech in spatially separated noise may not be the main driving factor for implantation. It is possible that improved localisation, quality of life or tinnitus suppression may be the main goals. As no improvement over the 24-month post-operative period was observed for any of these measures in the current study, we were able to estimate the post-op test–retest variability (or standard deviation) for each measure. Twice the standard deviation gives a 95% confidence interval for a ‘true’ difference in scores for an individual patient, allowing us to present the following chances of significant improvement in Table 4 to prospective patients.

There was a larger variability in results observed for tinnitus; therefore, these probabilities can only be claimed at an 84% level of certainty, rather than 95% as demonstrated for SRT, localisation and SSQ.

#### 4.2.7. Participant Numbers

The current study enrolled 41 participants; however, full data sets were not available for each test measure. The 12-month data point was used for most analyses due to the two-way ANOVAs showing no significant difference between the 3-, 12- and 24-month results, and the 12-month point was also selected as it allowed the recipient sufficient time to adjust to their cochlear implant. One participant became a non-user after 3 months; therefore, there was a maximum of 40 data points available for all tests at the 12-month point. Please refer to Table 5 for the explanation for different subject numbers for each test.

## 5. Discussion

The majority of recipients in the current study demonstrated significant improvement in their speech perception performance after receiving a CI. The group results for spatially separated SRT showed significantly better results when noise was presented both to the implanted ear and the contralateral ear, although a large amount of individual variability was observed. The average CVC phoneme score for the implanted ear was not significantly different from the mean score obtained from CI recipients with bilateral hearing loss at the Melbourne Cochlear Implant Clinic [27], although the difference was approaching significance. This may be interpreted as a trend towards slightly poorer scores for speech perception in quiet for this patient cohort than the standard CI population, but it may not have any clinical implications for the patient themselves because, typically in these situations, the dominant ear would be sufficient.

The results of the current study were consistent with the literature and showed significant improvements post-operatively as a group for localisation, but with considerable variability. No further improvements were seen from the 12- to the 24-month point, which is consistent with reports by Buss (2018) and Dillon (2017) but contrasts with those of Sullivan (2020), who continued to see a trend for improvement up to 6 years post-implant, and Thompson (2022), who saw improvements between the one- and five-year follow-up appointments. Sullivan suggests that years of auditory rehabilitation may be necessary for patients to reach their full potential [3,32,33,34].

Many studies have shown that a CI has the potential to help ease a recipient’s tinnitus severity, or to at least make them less aware of the tinnitus by the introduction of another stimulus. The results of the current study are consistent with many in the literature showing a significant reduction in reported tinnitus post-operatively for our patient cohort, but there is a large amount of variability between patients [2,3,4,5,35]. We did not observe any further improvements beyond the 12-month point.

The results of the current study regarding duration were consistent with those of Tavora and Boisvert [17], indicating a lack of correlation with SRT, SSQ, localisation, tinnitus or the IOI-HA. Similarly, Lindquist et al. (2023) found no difference in outcome measures for recipients with a duration of SSD greater than 10 years compared to a duration of less than 10 years [35]. It has been suggested that monaural auditory stimulation may be sufficient to maintain the integrity of the auditory pathways bilaterally [36], and indeed, the test measures mentioned above rely on binaural, real-life listening as a result of bilateral stimulation. The duration of loss in the current study was correlated, however, with the phoneme score for the implanted ear, with those patients with a shorter duration obtaining a higher score. There was one recipient in the current study with a congenital onset of asymmetric deafness. This recipient did not receive any meaningful auditory stimulation from the cochlear implant, which is likely a reflection of the re-organisation of the auditory system during the sensitive period [37,38].

Whilst the current study did not show any correlation between the duration of hearing loss and any binaural outcome measures, those recipients with a duration of asymmetrical loss greater than five years did demonstrate a trend towards overall lower satisfaction scores on the IOI-HA. It is possible that those recipients had adjusted somewhat to their hearing loss and felt that the implant did not meet their expectations in terms of restoring binaural hearing.

The results from the current study show a non-use rate at 12 months of 7%, which increased to 13% at the 24-month point. Three of these recipients achieved significant phoneme scores, suggesting that the decision to no longer wear the processor was not necessarily related to a lack of objective benefit but, rather, to a lack of subjective benefit, as highlighted by the low IOI-HA scores obtained by these recipients. For CI recipients with unilateral, or asymmetrical, hearing loss, objective results may not be the best measure of a successful outcome. Quality of life measures may be a more sensitive tool, as a measured improvement in speech perception in a sound-proof booth may not equate to real-life listening benefits for that particular recipient. For the group of recipients in the current study, speech perception scores were not correlated with the SSQ, but sound localisation was correlated with domain 2 ‘Spatial Hearing’, suggesting that the SSQ may be a meaningful way to measure the real-life benefits of improved localisation. The IOI-HA is a quick measure of overall device satisfaction and was correlated with SRT and with device use. Sound processor usage for the entire group was correlated with speech in noise scores, with those recipients who gained the most improvement wearing their processor longer each day.

The non-use rates in the current study appear slightly higher than those reported by other studies, which varied from 3.7% to 4.4% for studies that followed recipients for over 12 months [2,5,6], but are similar to the reported long-term non-use rate for bilaterally deaf CI users [19]. Whilst the current study did not employ a strict rehabilitation program, elective non-use by those patients who did gain a benefit in speech perception appears to be related to limited benefit gained in noisy situations, which is often the environment where most patients had hoped to gain the most improvement. A possible impact of the COVID-19 pandemic was also observed, with five participants unwilling to attend their 24-month appointment. Recipient reports often mentioned that while working from home and without complex social listening environments, they often elected not to wear the processor.

The results from the current study, across all domains, are consistent with those in the literature reporting large amounts of variability in outcomes [3,35], emphasising the importance of the discussion of expectations and motivation in the pre-operative period [17]. Indeed studies show that cochlear implant recipients with unilateral hearing loss require tailored care, focusing on candidacy, counselling and rehabilitation [39]. Considering these issues, the results from the current study have been used to develop a preliminary, unvalidated counselling tool for clinicians to use when discussing potential implantation with prospective cochlear implant recipients. Please see the Supplementary Material for a copy of the tool.

Patients with asymmetrical hearing loss often present to the clinic with complaints of difficulty hearing in noise, issues hearing from the ‘deaf’ side, tinnitus concerns and a lack of localisation. These topics were directly addressed in the tool. Whilst the group averages in the current study showed significant improvement in all domains measured, the variability in outcomes within the group makes it difficult for a clinician to predict the outcomes for an individual candidate. Being able to directly counsel a prospective recipient on their chances of improvement in each of the listening situations may help them reach a decision on implantation.

When discussing the ability to understand speech in quiet with the cochlear implant, our results suggest that this is equivalent to patients with bilateral hearing loss who undergo implantation.

For patients whose main concern is listening on their ‘deaf’ side, SRT testing in spatially separated noise demonstrated that 45% of patients showed significant improvement, 52.5% showed no change, and 2.5% were significantly worse. In terms of localisation, 27% of patients showed a significant improvement, 66% showed no significant change, and 7% were significantly worse post-op. In terms of tinnitus, 34% of patients showed significant improvement, 58% showed no significant change, and 8% were significantly worse. These figures can be used to address the patient’s main goals for the CI.

Counselling sessions must also revolve around the subjective benefits of implantation for this group. Whilst we can inform on the chances of improvement in each of the objective measures, these results do not always correlate with subjective benefit as measured by the IOI-HA and the SSQ. Whilst we saw significant post-operative improvements for the SSQ overall, and for each individual domain, looking at the distribution of responses, 42% of patients indicated significant improvement, 52% showed no change, and 6% of patients indicated a significantly worse score. The SSQ was correlated with the SRT, localisation, tinnitus and speech processor use but not with speech in quiet. Indeed, for the five non-users at the 24-month point, CVC phoneme scores ranged from 0% up to 70%. Two of the most important questions on the IOI-HA related to whether the patient felt the entire process was worth it. Question 2 of the questionnaire was ‘How much has the hearing aid (CI) helped in the situation you most wanted to hear better?’, and while 56% of patients said it helped ‘quite a lot’ or ‘very much’, 13% of patients said it helped ‘not at all’. This corresponds to our reported 24-month non-use rate of 24%. In response to question four ‘How much was the hearing aid (CI) worth the trouble?’, 75% of participants said it was either ‘quite a lot’ or ‘very much’ worth it; however, 6% of patients said it was ‘not at all’ worth it.

We can advise patients that those with a shorter duration of hearing loss in the ear to be implanted tend to have better overall satisfaction with their device, but duration was not correlated with speech in noise, tinnitus or localisation outcomes. For these reasons, we have suggested clinical guidelines for a duration of less than 20 years for potential recipients with unilateral hearing loss. This 20-year guideline is based on the observed trend of lower IOI-HA satisfaction and not on binaural objective measures. Each potential recipient, however, is treated on a case-by-case basis, and a duration longer than this can be considered if the patient understands the risks and is highly motivated to proceed with implantation.

While the counselling tool developed in this study appears promising, it is derived from a small patient cohort and requires further validation with wider populations. It does, however, offer clinicians some guidance when counselling prospective patients to a decision regarding implantation that best meets their goals. Any specific difficulties experienced by the patient can be discussed in terms of likelihood of improvement, with the understanding that objective outcomes are highly variable and not always correlated with perceived subjective benefit.

## 6. Limitations and Future Research

The current study provided a report on subjective and objective outcome measures from a group of 41 adult cochlear implant recipients with unilateral hearing loss from the Melbourne Cochlear Implant Clinic. It is important to note, however, that this was a prospective, single-site study that would have been more powerful had participant numbers been higher. The power would also have been increased had there been less variability in the duration of hearing loss. Having such variability did, however, allow correlations to be made between outcome measures and the duration of hearing loss. A degree of participant selection bias was also likely involved, as only motivated individuals with the capacity to attend extra appointments agreed to take part in the project.

The speech perception results for the current study focused on CI alone speech in quiet and binaural spatially separated speech in noise to investigate absolute CI speech perception and the impact of cochlear implantation on the head-shadow effect. Binaural squelch and summation were not considered in the study design, but the addition of front-on speech and noise testing would have provided a more complete picture of true binaural hearing restoration provided by the cochlear implant. The ability of a cochlear implant to overcome binaural hearing deficits for patients with unilateral hearing loss would have been further validated had a control group receiving a CROS or BAHA device been included in the study design, as these devices provide the ability to overcome the headshadow effect but do not facilitate restoration of binaural hearing cues.

Further investigation is warranted into device non-use by recipients who gained significant speech perception benefit from their cochlear implant. The results from the current study showed that three non-users at the 12-month point demonstrated significant phoneme scores with their device but indicated low satisfaction on the IOI-HA questionnaire. Objective speech perception results are likely not the most sensitive outcome measure for this patient cohort. Recipient comments regarding a reduction in complex listening situations as a result of the COVID-19 pandemic may have been a contributing factor to an increase in device non-use and in fact resulted in five participants being unwilling to attend their 24-month appointment. Further qualitative research into lifestyle or personal reasons that particular recipients elect not to persist with device use, irrespective of objective results, is warranted to better understand this issue of elective non-use.

Future validation of the counselling tool developed in this study may help with the counselling of these patients and hopefully help minimise issues of non-use.

## 7. Conclusions

Cochlear implantation for patients with asymmetrical hearing loss has the potential to offer improvements in head-shadow effects, sound localisation, and tinnitus. Subjective measures show significant improvements post-operatively. The large amount of variability in outcomes, however, means that the counselling aspect of the pre-operative work-up is imperative. Incorporating the evidence-based counselling tool developed from the results of the current study can help guide prospective recipients through their decision-making process, enabling them to make a fully informed decision regarding implantation based upon realistic expectations of outcomes.

## Figures and Tables

**Table 1:** Participant demographics.

Demographic	Average	Range
Age at Implantation (YRS)	53.2	20.7–76.5
Duration of Hearing Loss (YRS)	5.8	0.3–21 (41*)
Gender	Male: 20	Female: 21
PTA: CI Ear	98.2 dB	68–120 dB
PTA: Contra Ear	23.9 dB	5–53.8 dB
True SSD Contra EarPTA <20 dB	*n* = 21	5–18.75 dB
Unilateral Loss ContraTA >20 dB	*n* = 20	25–120 dB

**Table 2:** Group SSQ results from pre-operative to 12 months post-operative and significance of the difference.

	Pre-Operative Score		12 m Post-Operative Score		Significance (α = 0.0125)	Confidence Interval	Effect Size
	Mean	SD	Mean	SD	*p*		
SSQ Total Score	13.65	4.417	16.588	4.884	<0.001	−4.664 to −1.885	0.836
Domain 1 Speech Hearing	4.272	1.559	4.564	1.969	<0.001	−1.763 to −0.622	0.741
Domain 2 Spatial Hearing	3.36	1.796	4.736	2.125	<0.001	−1.956 to −0.796	0.842
Domain 3 Qualities of Hearing	5.944	1.688	6.634	1.569	=0.005	−1.154 to −0.226	0.527

**Table 3:** Sound processor use (hours per day).

	Mean	Standard Deviation	Range
3 months post-operative (*n* = 41)	9.14	3.729	1.4–14.4
12 months post-operative (*n* = 41)	8.29	4.564	0–14.4
24 months post-operative (*n* = 36)	7.99	4.736	0–18

**Table 4:** Probabilities of significant improvement post-CI.

	SRT	Tinnitus	Localisation	SSQ
Chance of Significant Improvement	45.0%	34.0%	27.0%	42.0%
Chance of No Significant Change	52.5%	58.0%	66.0%	52.0%
Chance of Significant Deterioration	2.5%	8.0%	7.0%	6.0%

**Table 5:** Participant numbers for each test measure at 12 months post-operative.

Test at 12 Months Point	Number	Explanation
SRT S90N270	39	1 non-user, 1 participant too fatigued to continue
SRT S270N80	40	1 non-user
Localisation	39	1 non-user, 1 participant did not complete pre-op
Tinnitus	40	1 non-user, 2 participants did not complete pre-op
SSQ	33	1 non-user SSQ introduced after 1st 4 participants completed pre-op; therefore, only 37 had pre and post available 3 participants did not return questionnaire
IOI-HA	32	1 non-user IOI-HA introduced only after 1st 5 participants completed pre-op 3 participants did not return questionnaire
Data Usage (24 months)	36	5 participants unwilling to attend 24-month apt due to COVID-19 pandemic

## Data Availability

The data presented in this study are owned by the Eye and Ear Hospital and are therefore not publicly available due to privacy.

## Supplementary Materials

The following supporting information can be downloaded at: https://www.mdpi.com/article/10.3390/audiolres16040102/s1. Supplementary Material File: Guidelines for Recommending Cochlear Implantation in Adults with Single Sided Deafness.
