# Supplementary material for: Results for Adults with Asymmetric Hearing Loss Who Receive a Cochlear Implant
Source: Audiol Res. 2026 Jul 23;16(4):102. doi: 10.3390/audiolres16040102 (PMC13509795; doi:10.3390/audiolres16040102)
Supplement: Supplementary file 1 [file audiolres-16-00102-s001.zip › audiolres-4332976-supplementary.pdf]

GUIDELINES FOR RECOMMENDING COCHLEAR IMPLANTATION IN ADULTS WITH SINGLE SIDED DEAFNESS

The following information may assist you when considering if cochlear implantation is an option for improving hearing and communication for a person who has a severe-to-profound hearing loss in one ear and a mild hearing loss or normal hearing in the other ear.

Audiometric guidelines:

A cochlear implant is likely to improve detection of sound if, in the ear considered for implantation:

- Unaided PTA is 70dBHL or worse in the worse hearing ear
- Duration of hearing loss is <20 years

Can also refer to audiogram on the right for highlighted hearing range.

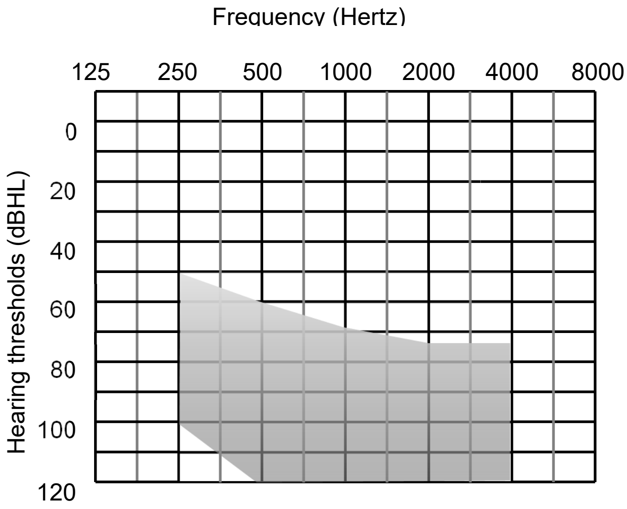

Speech perception guidelines:

Patients are encouraged to trial a CROS hearing aid and/or a Baha device on a softband, then undergo speech perception in adaptive noise (SRT) testing. A cochlear implant could provide improved hearing if:

- There is less than 2dB improvement in SRT with trial CROS/Baha with speech directed to the bad hearing ear and noise directed to the good ear. This is based on the first quartile SRT improvement distribution from the study cohort

Guidelines for expectations:

Patients with a duration of hearing loss <5 years likely to score better on CVC phonemes and be more satisfied with CI.

Patients understand that:

- If seeking improved **hearing in noise**, approx. 45% chance of improvement and 3% chance of getting worse
- If seeking improvement in **localisation**, approx. 27% chance of improvement and 7% chance of getting worse
- If seeking reduction in **tinnitus**, approx. 34% chance of improvement and 8% chance it becomes worse

Medical and developmental guidelines:

Cochlear implantation is an option if:
• Normal inner ear and retro-cochlear anatomy<br>• The surgical procedure can be performed with minimal risk to the patient<br>• Evidence that the patient has sufficient cognitive ability that they understand the potential benefits and limitations of cochlear implantation
